# Association of maternal obesity with preterm birth phenotype and mediation effects of gestational diabetes mellitus and preeclampsia: a prospective cohort study

**DOI:** 10.1186/s12884-022-04780-2

**Published:** 2022-06-01

**Authors:** Kan Liu, Yixuan Chen, Jianing Tong, Aiqi Yin, Linlin Wu, Jianmin Niu

**Affiliations:** Department of Obstetrics, Shenzhen Maternity & Child Healthcare Hospital, The First School of Clinical Medicine, Southern Medical University, Shenzhen, Guangdong China

**Keywords:** Obesity, Prepregnancy BMI, Preterm birth, Phenotype, Gestational diabetes mellitus, Preeclampsia

## Abstract

**Background:**

The association between maternal obesity and preterm birth remains controversial and inconclusive, and the effects of gestational diabetes mellitus (GDM) and preeclampsia (PE) on the relationship between obesity and preterm birth have not been studied. We aimed to clarify the relationship between prepregnancy body mass index (BMI) and the phenotypes of preterm birth and evaluate the mediation effects of GDM and PE on the relationship between prepregnancy BMI and preterm birth.

**Methods:**

We conducted a prospective cohort study of 43,056 women with live singleton births from 2017 through 2019. According to the WHO International Classification, BMI was classified as underweight (BMI < 18.5 kg/m2), normal weight (BMI 18.5–25 kg/m2), overweight (BMI 25–30 kg/m2) and obese (BMI ≥30 kg/m2). Preterm birth was defined as gestational age less than 37 weeks (extremely, < 28 weeks; very, 28–31 weeks; and moderately, 32–36 weeks). The clinical phenotypes of preterm birth included spontaneous preterm birth (spontaneous preterm labor and premature rupture of the membranes) and medically indicated preterm birth. We further analyzed preterm births with GDM or PE. Multivariable logistic regression analysis and causal mediation analysis were performed.

**Results:**

Risks of extremely, very, and moderately preterm birth increased with BMI, and the highest risk was observed for obese women with extremely preterm birth (OR 3.43, 95% CI 1.07–10.97). Maternal obesity was significantly associated with spontaneous preterm labor (OR 1.98; 95% CI 1.13–3.47), premature rupture of the membranes (OR 2.04; 95% CI 1.08–3.86) and medically indicated preterm birth (OR 2.05; 95% CI 1.25–3.37). GDM and PE mediated 13.41 and 36.66% of the effect of obesity on preterm birth, respectively. GDM mediated 32.80% of the effect of obesity on spontaneous preterm labor and PE mediated 64.31% of the effect of obesity on medically indicated preterm birth.

**Conclusions:**

Maternal prepregnancy obesity was associated with all phenotypes of preterm birth, and the highest risks were extremely preterm birth and medically indicated preterm birth. GDM and PE partially mediated the association between obesity and preterm birth.

**Supplementary Information:**

The online version contains supplementary material available at 10.1186/s12884-022-04780-2.

## Background

Preterm birth is a crucial global public health issue. Globally, preterm birth has been found to affect an estimated 10.6% of livebirths [1]. In China, the incidence rate of preterm birth has increased over the past three decades, and it was found to be approximately 7% [2]. Prematurity is the leading cause of morbidity and mortality in children younger than 5 years [3] and ranks first among the causes of perinatal mortality in China [4]. In view of the perniciousness of preterm birth, the identification of risk factors for preterm birth is imperative, especially in developing countries.

Maternal obesity is a growing public health problem worldwide, and it is the most common medical condition in women of reproductive age. Obesity causes adverse consequences for both mothers and their children. Given the high prevalence and associated risks, maternal obesity has been identified as the most important preventable risk factor for adverse pregnancy outcomes in many countries.

Prepregnancy obesity has been found to be associated with preterm birth; however, the relationship remains controversial and inconclusive in the literature. Some previous studies have found a positive correlation between obesity and preterm birth [5, 6], while others have not [7]. In addition, the relationship differs in different subgroups classified by gestational age and clinical phenotypes. Maternal obesity was associated with a lower risk of spontaneous preterm birth [8], whereas prepregnancy body mass index (BMI) was not associated with the risk of spontaneous preterm labor before 32 weeks of gestation [9]. In general, maternal obesity increases the risk of gestational diabetes and hypertensive disorders of pregnancy, which may lead to medically indicated preterm birth. Khatibi et al. reported that among women in all obesity categories, there was no association with medically indicated preterm birth [10]. Additionally, previous studies have relied on retrospectively collected data and/or self-reported BMI thus are prone to misclassification or recall bias.

Given the above controversial, inconclusive results and limitation, we performed a prospective cohort study to explore the relationships between prepregnancy BMI and the phenotypes of preterm birth. Maternal obesity has been found to be related to gestational diabetes mellitus (GDM) and preeclampsia (PE), and these two diseases are important maternal factors for preterm birth [9]; however, the effects of GDM and PE on the relationship between obesity and preterm birth have not been studied. For the first time, we studied the mediation effects of GDM and PE on the relationship between BMI and preterm birth.

## Methods

### Study population

Birth Cohort in Shenzhen (BiCoS), conducted by Shenzhen Maternity & Child Healthcare Hospital, is a prospective observational birth cohort study. We recruited participants with a singleton pregnancy at gestational age of 6–8^+ 6^ weeks who consented to participate in the cohort from 2017 to 2019. Postmature birth was excluded. All participants signed written, informed consent and agreed to be contacted for future studies. Our obstetric professional research assistants collected a comprehensive set of data on pregnancy including maternal characteristics, maternal risk factors and pregnancy complications at every antenatal clinic visit. After giving birth, delivery information and neonatal outcomes were abstracted from medical records. Quality control on this dataset included procedures for monthly quality checks and an annual secondary review of 10–15% of the records.

A total of 43,056 pregnancies were available for inclusion in this study. We excluded 860 (2%) pregnancies from the dataset for the following reasons: missing maternal prepregnancy BMI, prepregnancy hypertension or diabetes, cervical incompetence, still birth and neonatal abnormalities.

### Study variables

The primary explanatory variable was maternal prepregnancy BMI (weight in kilograms/height in meters2). We used the World Health Organization International Classification of BMI [11]. The categories were underweight (BMI < 18.5 kg/m2), normal weight (BMI 18.5- < 25 kg/m2), overweight (BMI 25- < 30 kg/m2) and obese (BMI ≥ 30 kg/m2). The normal prepregnancy BMI group was the reference group. Height (to the nearest 0.1 cm) and weight (to the nearest 0.1 kg) were measured twice with the subjects at the first perinatal visit (6–8^+ 6^ gestational weeks) in light clothing and without shoes, and were obtained using a stadiometer and scale with calibrated electronic scale. Prepregnancy BMI was calculated using the above values. Information about GDM or PE was classified by the woman’s physician at the time of hospital discharge. We also included the following risk factors in the analysis: maternal age, education level, nullipara, parity and assisted reproductive technology.

### Definition of outcome

Preterm birth was defined as delivery occurring before 37 weeks of gestation. Gestational age was based on the last menstrual period if the last menstrual period and the earliest ultrasound estimate were within 10 days of each other. If not, the earliest ultrasound evaluation was used to define gestational age. We subdivided the outcome into three groups by gestational age: extremely (< 28 weeks gestation), very (28–31 weeks gestation), and moderately (32–36 weeks gestation) premature [12]. The clinical phenotypes of preterm birth included spontaneous preterm labor, premature rupture of the membranes and medically indicated preterm birth [13]. Furthermore, we categorized preterm births into PE and GDM groups. The diagnosis of PE was based on the Chinese Medical Association of Obstetricians and Gynecologists (2015) guidelines on hypertension during pregnancy (with reference to foreign guidelines [14]), while GDM was based on Chinese Medical Association of Obstetricians and Gynecologists (2015) guidelines on gestational diabetes (with reference to HAPO study [15] and foreign guidelines [16]).

### Statistical analysis

Continuous variables are reported as the mean ± standard deviation (SD) and were compared using Student’s independent t-test or Mann-Whitney U test depending on the normality assumption. Categorical variables are presented as numbers and percentages and were compared using the chi-square test or Fisher’s exact test (if an expected value ≤5 was found). A multivariate logistic regression model was used to calculate odds ratios (ORs) with 95% confidence intervals (CIs) of BMI for preterm birth after adjusting for maternal age, educational level, and assisted reproductive technology. Relative risk (RR) was used to investigate the preterm birth risk in participants with or without GDM/PE. The above analyses were performed using IBM SPSS Version 25 (SPSS Statistics V25, IBM Corporation, Somers, New York). Furthermore, causal mediation analysis was used to estimate the OR for the natural direct effect (NDE) and the natural indirect effect (NIE) of preterm birth mediated through GDM or PE. We also estimated the proportion of the effect mediated to reflect the extent of mediation, where 100% indicates that all of the total effect is mediated and 0% indicates that there is no mediation. These analyses were performed by using R statistical software version 3.5.2 with the package ‘mediation’. The statistical significance level for all the tests was set at a two-tailed *P*-value < 0.05.

## Results

### Characteristics of the study population

Information from 43,056 singleton births was collected for the cohort study between 2017 and 2019. We excluded 860 (2%) births for the following reasons: missing prepregnancy BMI data (397), preexisting diabetes or hypertension (281), cervical incompetence (94), stillbirths and neonatal abnormalities (88). The final study population included 42,196 deliveries of live singleton infants, including 2768 (6.56%) preterm births. The average age of all participants was 31.26 ± 4.46 years, the prepregnancy BMI was 21.08 ± 2.84, and the parity was 0.60 ± 0.62. Most participants had a bachelor’s degree (65.49%), and only 1381 (3.27%) became pregnant through assisted reproductive technology. The participants who experienced preterm birth had a significantly higher maternal age and assisted reproductive technology rate and lower educational level (*P* < 0.001). Compared with women with full-term births, those with preterm births had a significantly higher rate of GDM or PE (21.46% vs. 15.70 and 10.44% vs. 1.00% for GDM and PE, respectively, *P* < 0.001) (Table 1).

**Table 1:** Characteristics of 42,196 Singleton Deliveries in Shenzhen 2017–2019

Parameters	Full-term birth (*n* = 39,428)	Preterm birth (*n* = 2768)	All (*n* = 42,196)	P
Maternal age (years), mean ± SD	31.22 ± 4.43	31.80 ± 4.86	31.26 ± 4.46	**< 0.001**
Maternal age group				**< 0.001**
< 35 years, n (%)	30,029 (76.16%)	1942 (70.16%)	31,971 (75.77%)	
≥ 35 years, n (%)	9399 (23.84%)	826 (29.84%)	10,225 (24.23%)	
Pre-pregnancy BMI (kg/m^2^), mean ± SD	21.06 ± 2.82	21.47 ± 3.13	21.08 ± 2.84	**< 0.001**
Pre-pregnancy BMI group ^a^				**< 0.001**
Underweight, n (%)	6479 (16.43%)	431 (15.57%)	6910 (16.38%)	
Normal weight, n (%)	29,606 (75.09%)	1993 (72.00%)	31,599 (74.89%)	
Overweight, n (%)	2996 (7.60%)	299 (10.80%)	3295 (7.81%)	
Obesity, n (%)	347 (0.88%)	45 (1.63%)	392 (0.93%)	
Educational levels				**< 0.001**
High school, n (%)	8317 (24.36%)	689 (29.57%)	9006 (24.69%)	
Bachelor, n (%)	22,425 (65.69%)	1458 (62.58%)	23,883 (65.49%)	
Master, n (%)	3398 (9.95%)	183 (7.85%)	3581 (9.82%)	
Nulliparous				0.068
No, n (%)	2137 (5.45%)	173 (6.28%)	2310 (5.51%)	
Yes, n (%)	37,040 (94.55%)	2581 (93.72%)	39,621 (94.49%)	
Parity, mean ± SD	0.60 ± 0.61	0.62 ± 0.63	0.60 ± 0.62	0.284
Parity group				0.344
0, n (%)	17,851 (46.16%)	1244 (45.47%)	19,095 (46.11%)	
1, n (%)	18,688 (48.32%)	1319 (48.21%)	20,007 (48.31%)	
2–3, n (%)	2110 (5.46%)	170 (6.21%)	2280 (5.51%)	
≥ 4, n (%)	27 (0.07%)	3 (0.11%)	30 (0.07%)	
ART				**< 0.001**
No, n (%)	38,203 (96.89%)	2612 (94.36%)	40,815 (96.73%)	
Yes, n (%)	1225 (3.11%)	156 (5.64%)	1381 (3.27%)	
GDM				**< 0.001**
No, n (%)	33,236 (84.30%)	2174 (78.54%)	35,410 (83.92%)	
Yes, n (%)	6192 (15.70%)	594 (21.46%)	6786 (16.08%)	
PE				**< 0.001**
No, n (%)	39,034 (99.00%)	2479 (89.56%)	41,513 (98.38%)	
Yes, n (%)	394 (1.00%)	289 (10.44%)	683 (1.62%)	

*Abbreviations*: *BMI* Body mass index, *ART* Assisted reproduction technology, *GDM* Gestational diabetes mellitus, *PE* Preeclampsia

Significant *p*-values are emphasized in bold font

^a^ underweight (BMI < 18.5 kg/m2), normal weight (BMI 18.5–25 kg/m2), overweight (BMI 25-30 kg/m2), (BMI ≥30 kg/m2)

### BMI and risks of preterm birth by gestational age

The association between prepregnancy BMI and preterm birth according to different gestational ages was investigated, and the findings are presented in Table 2. Compared with prepregnancy normal weight, maternal overweight (adjusted OR 1.42; 95% CI [1.23–1.63]) and obesity (2.01 [1.44–2.82]) were significantly associated with increased risks of overall preterm birth. When preterm birth was subdivided into three subgroups by maternal age, maternal overweight was significantly associated with an increased risk of only moderately preterm birth (1.46 [1.26–1.69]). In the obesity category, the risk of preterm birth increased with decreasing gestational age (1.87 [1.29–2.73], 2.52 [1.11–5.74]) and (3.43 [1.07 to 10.97]) for moderately, very and extremely preterm births, respectively). Maternal underweight was associated with slightly increased risks of overall preterm birth (1.06 [0.94 to 1.19]) and moderately preterm birth (1.09 [0.96 to 1.24]).

**Table 2:** Maternal pre-pregnancy BMI and risks of preterm birth by gestational age

**Parameters**	**Event/N (%)**	**Preterm birth**		**Event/N (%)**	**Extremely preterm birth** ^**a**^	
		**OR (95% CI)**	**P**		**OR (95% CI)**	**P**
Pre-pregnancy BMI group			< 0.001			0.072
Normal weight (18.5–24.9)	1993/31599 (6.31%)	ref.	–	80/29686 (0.27%)	ref.	–
Underweight (< 18.5)	431/6910 (6.24%)	1.06 (0.94 to 1.19)	0.339	13/6492 (0.20%)	0.70 (0.37 to 1.33)	0.281
Overweight (25.0–29.9)	299/3295 (9.07%)	**1.42 (1.23 to 1.63)**	**< 0.001**	11/3007 (0.37%)	1.42 (0.75 to 2.69)	0.28
Obesity (≥30.0)	45/392 (11.48%)	**2.01 (1.44 to 2.82)**	**< 0.001**	3/350 (0.86%)	**3.43 (1.07 to 10.97)**	**0.038**
	**Event/N (%)**	**Very preterm birth** ^**a**^		**Event/N (%)**	**Moderately preterm birth** ^**a**^	
		**OR (95% CI)**	**P**		**OR (95% CI)**	**P**
Pre-pregnancy BMI group			0.156			< 0.001
Normal weight (18.5–24.9)	242/29848 (0.81%)	ref.	–	1671/31277 (5.34%)	ref.	–
Underweight (< 18.5)	47/6526 (0.72%)	0.96 (0.67 to 1.36)	0.8	371/6850 (5.42%)	1.09 (0.96 to 1.24)	0.171
Overweight (25.0–29.9)	27/3023 (0.89%)	1.12 (0.73 to 1.72)	0.594	261/3257 (8.01%)	**1.46 (1.26 to 1.69)**	**< 0.001**
Obesity (≥30.0)	6/353 (1.70%)	**2.52 (1.11 to 5.74)**	**0.027**	36/383 (9.40%)	**1.87 (1.29 to 2.73)**	**0.001**

*Abbreviations*: *BMI* Body mass index, *OR* Odds ratio, *CI* Confidence interval

Significant associations and *p*-values are emphasized in bold font

ORs were adjusted for maternal age, educational level, and assisted reproduction technology

^a^ Extremely preterm birth (< 28 weeks’ gestation), Very preterm birth (28–31 weeks’ gestation), Moderately preterm birth (32–36 weeks’ gestation)

### BMI and risks of preterm birth by clinical phenotype

As indicated in Table 3, maternal obesity was a significant predictor of all clinical phenotypes of preterm birth. Compared with normal weight, the adjusted ORs for preterm birth among obese women were as follows: spontaneous preterm labor (1.98 [1.13–3.47]), premature rupture of the membranes (2.04 [1.08–3.86]) and medically indicated preterm birth (2.05 [1.25–3.37]). Maternal overweight was significantly associated with premature rupture of the membranes (1.62 [1.26–2.09]) and medically indicated preterm birth (1.46 [1.19–1.80]). Maternal underweight was associated with slightly increased risks of spontaneous preterm labor (1.19 [0.99 to 1.43]) and premature rupture of the membranes (1.17 [0.94 to 1.47]) but was associated with a slightly decreased risk of medically indicated preterm birth (0.87 [0.72 to 1.07]). Finally, we estimated the risks of preterm birth after excluding pregnant women with GDM or PE (Table S1). Compared with the results demonstrated in Tables 2 and 3, we found the following results: 1) When women with GDM were excluded, the obesity-related risks of overall preterm birth remained unchanged (2.01 [1.44 to 2.82] vs 2.13 [1.39 to 3.28]), while the adjusted ORs of obesity for spontaneous preterm labor (1.86 [0.87–3.97]) and premature rupture of the membranes (2.20 [0.97–5.00]) were not statistically significant. 2) When women with PE were excluded, the obesity-related risks of overall preterm birth were slightly decreased (2.01 [1.44 to 2.82] vs 1.72 [1.17 to 2.53]), and the obesity-related risks of medically indicated preterm birth were significantly reduced (2.05 [1.25–3.37] vs 1.33 [0.65–2.69]).

**Table 3:** Maternal pre-pregnancy BMI and risks of clinical phenotypes of preterm birth

**Parameters**	**Event/N (%)**	**Spontaneous preterm labor**		**Event/N (%)**	**Premature rupture of the membranes**	
		**OR (95% CI)**	**P**		**OR (95% CI)**	**P**
Pre-pregnancy BMI group			0.021			< 0.001
Normal weight (18.5–24.9)	694/30300 (2.29%)	ref.	–	496/30102 (1.65%)	ref.	–
Underweight (< 18.5)	173/6652 (2.60%)	1.19 (0.99 to 1.43)	0.072	110/6589 (1.67%)	1.17 (0.94 to 1.47)	0.159
Overweight (25.0–29.9)	82/3078 (2.66%)	1.21 (0.94 to 1.55)	0.138	82/3078 (2.66%)	**1.62 (1.26 to 2.09)**	**< 0.001**
Obesity (≥30.0)	14/361 (3.88%)	**1.98 (1.13 to 3.47)**	**0.017**	12/359 (3.34%)	**2.04 (1.08 to 3.86)**	**0.029**
	**Event/N (%)**	**Medically indicated preterm birth**				
		**OR (95% CI)**	**P**			
Pre-pregnancy BMI group			< 0.001			
Normal weight (18.5–24.9)	803/30409 (2.64%)	ref.	–			
Underweight (< 18.5)	148/6627 (2.23%)	0.87 (0.72 to 1.07)	0.182			
Overweight (25.0–29.9)	135/3131 (4.31%)	**1.46 (1.19 to 1.80)**	**< 0.001**			
Obesity (≥30.0)	19/366 (5.19%)	**2.05 (1.25 to 3.37)**	**0.005**			

*Abbreviations*: *BMI* Body mass index, *OR* Odds ratio, *CI* Confidence interval

Significant associations and p-values are emphasized in bold font

ORs were adjusted for maternal age, educational level, and assisted reproduction technology

### Mediation effect of GDM/PE on the relationship between BMI and preterm birth

As indicated in Table S2, compared with women without GDM or PE, the RRs of preterm birth were significantly higher among women with GDM (1.43 [1.31 to 1.56]) or PE (7.09 [6.44 to 7.80]), while the results were unchanged in different BMI categories. Obese women had the highest rate of GDM among women who experienced preterm labor (40% vs 34.11% vs 20.87% vs 13.46% for obesity, overweight, normal weight and underweight, respectively, *P* < 0.001). The same results were observed among women with PE (28.89% vs 17.73% vs 10.29% vs 4.18% for obesity, overweight, normal weight and underweight, respectively, *P* < 0.001) (Table S3).

The direct association between BMI and GDM/PE, GDM/PE and preterm birth were also confirmed using logistic regression. It was found that maternal obesity was significantly associated with the incidence of GDM (OR:1.15, 95% CI: 1.14 to 1.16; *P* < 0.001) and PE (OR:1.17, 95% CI: 1.15 to 1.20; *P* < 0.001); preterm birth was positively associated with GDM (OR:1.47, 95% CI: 1.33 to 1.61; *P* < 0.001) and PE (OR:11.55, 95% CI: 9.87 to 13.52; *P* < 0.001).

Given the above results, causal mediation analysis was conducted to investigate the mediation effect of GDM or PE on the relationship between BMI (6–8^+ 6^ gestational weeks) and preterm birth. As indicated in Table 4, GDM mediated the association between prepregnancy BMI and preterm birth by 13.41% after controlling for confounders. Similarly, PE mediated the association between prepregnancy BMI and preterm birth by 36.66%. Then, we conducted mediation analysis for the clinical phenotypes. The results indicate that GDM had a significant mediating effect on the association between BMI and spontaneous preterm labor (proportion mediated = 32.80%) and premature rupture of the membranes (proportion mediated = 12.80%). Finally, GDM and PE mediated 7.5 and 64.31% of the effect of obesity on medically indicated preterm birth, respectively, and PE mediated 4.51% of the effect of obesity on premature rupture of the membranes.

**Table 4:** Mediation effect of GDM/PE between maternal pre-pregnancy BMI and preterm birth

**Preterm birth**	**Mediator**	**Adjusted OR(95%CI)**			**Proportion mediated**
		**Natural direct effect (NDE)**	**Natural indirect effect (NIE)**	**Total effect (TE)**	
All preterm births	GDM	1.002 (1.001 to 1.003)***	1.0003 (1.0002 to 1.0005)***	1.002 (1.001 to 1.003)***	13.41%
Spontaneous preterm labor	GDM	1.0001 (0.9996 to 1.001)	1.0001 (1.00004 to 1.0002)***	1.0002 (0.9997 to 1.001)	32.80%
Premature rupture of the membranes	GDM	1.001 (1.0002 to 1.001)**	1.0001 (1.0000 to 1.0002)**	1.001 (1.0003 to 1.001)**	12.80%
Medically indicated preterm birth	GDM	1.001 (1.001 to 1.002)***	1.0001 (1.00003 to 1.0002)**	1.001 (1.001 to 1.002)***	7.50%
All preterm births	PE	1.001 (1.0005 to 1.002)***	1.001 (1.0001 to 1.001)*	1.002 (1.001 to 1.003)***	36.66%
Spontaneous preterm labor	PE	1.0003 (0.9997 to 1.001)	0.99997 (0.9999 to 1.000003)	1.0003 (0.9997 to 1.001)	0
Premature rupture of the membranes	PE	1.001 (1.0003 to 1.001)**	1.00004 (1.0000001 to 1.0001)*	1.001 (1.0003 to 1.001)**	4.51%
Medically indicated preterm birth	PE	1.0004 (0.9999 to 1.001)	1.001 (1.0002 to 1.001)*	1.001 (1.0004 to 1.002)***	64.30%

*Abbreviations*: *BMI* Body mass index, *GDM* Gestational diabetes mellitus, *PE* Preeclampsia, *OR* Odds ratio, *CI* Confidence interval

Continuous variable BMI was analyzed as independent variable in these causal mediation analyses

ORs were adjusted for maternal age, educational level, and assisted reproduction technology

Basically, the OR estimations were rounded to 3rd decimal. If the rounded result was 1.000, then the result would be revised and rounded to the non-zero decimal digit

*, *P* < 0.05; **, *P* < 0.01; ***, *P* < 0.001

## Discussion

In previous studies, the association between prepregnancy obesity and clinical phenotypes of preterm birth was inconsistent and inconclusive. The reason underlying the modification of the effect of obesity on preterm birth by clinical phenotype is unknown. In this cohort study, we found that maternal prepregnancy overweight and obesity were associated with an increased risk of overall preterm birth. Obesity was associated with increased risks of preterm births at all gestational ages, while the highest risk was observed for extremely preterm births. On the other hand, women with a prepregnancy BMI in the obese category were at a significantly elevated risk for preterm birth related to spontaneous preterm labor, premature rupture of the membranes, or a medical indication, while the highest risk was observed for medically indicated preterm birth. Additionally, GDM and PE partially mediated the association between obesity and preterm birth. GDM primarily partially mediated the association between obesity and spontaneous preterm birth, while PE primarily partially mediated the association between obesity and medically indicated preterm birth.

In the present research, we noted that overweight and obesity were associated with increased risks of medically indicated preterm birth. Some previous investigations have suggested that obesity-related increased risks of medically indicated preterm deliveries may largely be due to obesity-related pregnancy disorders [17, 18]. Several studies have demonstrated that subclinical metabolic dysfunctions in obese women, such as GDM and PE, are associated with adverse pregnancy outcomes, including preterm birth [19, 20]. Obesity is a well-known risk factor for GDM and PE [21]. Liang et al. [6] demonstrated that women with prepregnancy obesity had a 3.7-fold increased risk of PE compared to women with normal prepregnancy BMIs. In addition, if all mothers had a normal prepregnancy BMI, 41.63% PE and 14.75% GDM could be avoided [22]. However, previous studies focused on the associations between obesity and GDM/PE as well as GDM/PE and preterm birth, and the mediation effect of GDM or PE on the relationship between obesity and preterm birth has not been well demonstrated. According to the mediation analysis in our study, we found that GDM and PE were partial mediators of the relationship between prepregnancy obesity and preterm birth. Moreover, the two diseases, especially PE, had an effect on the association of obesity with medically indicated preterm birth. This finding is in line with the result from a previous investigation [18], indicating that the excessive risk of obesity-related medically indicated preterm birth may be due to obesity-related pregnancy diseases. Finally, our results indicated that GDM principally partially mediated the association between obesity and spontaneous preterm birth.

With regard to spontaneous preterm birth, the results of previous studies have differed. Maternal overweight and obesity have been associated with increased, decreased, and neutral risks of preterm birth, and these associations have been debated in the literature. In our study, we found that maternal obesity was positively associated with spontaneous preterm labor and premature rupture of the membranes, while maternal overweight was positively associated with only premature rupture of the membranes. The mechanisms underlying the relationships between maternal obesity and adverse perinatal outcomes are complex. Although there is no unifying and definite mechanism responsible for the spontaneous preterm births associated with maternal obesity, on the basis of the available data, inflammation, endothelial dysfunction, insulin resistance, oxidative stress, and lipotoxicity seem to contribute to early placental and fetal dysfunction, which could further induce preterm birth. Pregravid obesity is associated with a systemic low-grade metabolic inflammatory state and subclinical endotoxemia [23]. Inflammation, which is related to both advanced maternal age and obesity, has been proposed as an important risk factor for preterm birth [24]. Spontaneous preterm births are associated with increased levels of inflammatory proteins (cytokines), such as interleukin 6, interleukin 1β, and tumor necrosis factor (TNF) α [25]. These cytokines are associated with cervical ripening and preterm myometrial contractions. Previous studies found that maternal obesity was associated with inflammatory upregulation through increased production of adipokines by adipose tissue [26]. An elevated inflammatory state may make obese women more prone to chorioamnionitis induced by subclinical infections such as genital and urinary tract infections, which may further enhance inflammation and increase the risk of spontaneous preterm birth [18]. Anne et al. showed a higher frequency of histologic chorioamnionitis in spontaneous preterm labor and premature rupture of the membranes [27]. Inflammation and infection account for 25 to 40% of all preterm births with intact membranes and 20 to 30% of cases of preterm premature rupture of membranes [28].

During pregnancy, obese women are more likely to have higher visceral fat mass, and increased visceral adipose mass is accompanied by decreased insulin sensitivity and elevated levels of glucose, which contribute to early placental and fetal dysfunction [29].

Consistent with the findings of previous studies [18, 30], we found that the risks of overall and moderately preterm birth were higher among underweight women, but the association was not statistically significant. In our study, maternal underweight was associated with slightly increased risks of spontaneous preterm labor and preterm premature rupture of membranes but slightly decreased risks of medically indicated preterm birth. The mechanisms underlying the different associations between underweight and the phenotype of preterm birth are unknown. We proposed that underweight women were prone to malnutrition, which could induce maternal and placental dysfunction. On the other hand, underweight women had fewer pregnancy disorders leading to fewer medically indicated preterm births.

To our knowledge, this is the first study conducted with BiCoS dataset. The strengths of our study include its large size, which allowed us to study the risks of preterm births by gestational age and clinical phenotype. Information on BMI was based on measured heights and weights, which is an advantage because self-reported weight is usually underestimated and self-reported height is usually overestimated. We measured height and weight at the first perinatal visit (6–8 + 6 gestational weeks) during which women have little change in their weight. Most importantly, we are the first to study the mediation effect of GDM or PE on the relationship between maternal prepregnancy BMI and preterm birth phenotype.

Although some confounding factors were controlled, alcohol consumption and maternal smoking were not adjusted because only 10 women in our data reported a history of alcohol consumption and smoking. Therefore, we did not adjust for those two variables.

However, there are a number of potential limitations of our study. First, we acknowledge that although we had a large cohort, the number of subjects was small in the obese category when we stratified preterm birth by gestational age and clinical phenotype. We were also unable to stratify the clinical phenotypes of preterm birth by gestational age. Similarly, when we conducted the mediation analysis, the sample size of each subgroup was limited. Additionally, although we attempted to adjust for potential confounding variables, we did not have information on variables such as other pre-existing medical conditions and previous history of preterm birth.

## Conclusions

In conclusion, our study notes that maternal prepregnancy obesity is an independent risk factor for all phenotypes of preterm birth. In addition, we conclude that GDM and PE partially mediate the association between prepregnancy obesity and preterm birth. Our findings support the potential importance of interventions to reduce prepregnancy obesity (such as reasonable diet and regular exercise) as an important strategy to reduce obesity-related pregnancy diseases and premature births.

## Supplementary Information

**Additional file 1: Table S1.** Maternal Pre-pregnancy BMI and Risks of Clinical Phenotypes of Preterm Birth without GDM/PE.

**Additional file 2: Table S2.** The relative risks of preterm birth with or without GDM/PE.

**Additional file 3: Table S3.** Distribution characteristics of GDM/PE in Different BMI Groups between Preterm Birth.

## Data Availability

The datasets used and analysed during the current study are available from the corresponding author on reasonable request.

## Abbreviations

BMI: Body mass index (BMI)
BiCoS: Birth Cohort in Shenzhen
CI: Confidence interval
GDM: Gestational diabetes mellitus
NDE: Natural direct effect
NIE: Natural indirect effect
OR: Odds ratio
RR: Relative risk
SD: Standard deviation
PE: Preeclampsia
