# Supplementary material for: Association of maternal obesity with preterm birth phenotype and mediation effects of gestational diabetes mellitus and preeclampsia: a prospective cohort study
Source: BMC Pregnancy Childbirth. 2022 Jun 1;22:459. doi: 10.1186/s12884-022-04780-2 (PMC9158369; doi:10.1186/s12884-022-04780-2)
Supplement: Supplementary file 1 — Additional file 1: Table S1. Maternal Pre-pregnancy BMI and Risks of Clinical Phenotypes of Preterm Birth without GDM/PE. [file 12884_2022_4780_MOESM1_ESM.docx]

**Table S1. Maternal Pre-pregnancy BMI and Risks of Clinical Phenotypes of Preterm Birth without GDM/PE**

|  | Preterm birth | |  | Spontaneous preterm labor | |  | Premature rupture of the membranes | |  | Medically indicated preterm birth | |
| --- | --- | --- | --- | --- | --- | --- | --- | --- | --- | --- | --- |
| Parameters | OR (95% CI) | P |  | OR (95% CI) | P |  | OR (95% CI) | P |  | OR (95% CI) | P |
| ***Participants without GDM*** |  |  |  |  |  |  |  |  |  |  |  |
| Pre-pregnancy BMI group |  | <0.001 |  |  | 0.104 |  |  | <0.001 |  |  | <0.001 |
| Normal weight (18.5-24.9) | ref. | - |  | ref. | - |  | ref. | - |  | ref. | - |
| Underweight (<18.5) | 1.07 (0.94 to 1.22) | 0.303 |  | 1.21 (0.99 to 1.48) | 0.058 |  | 1.22 (0.95 to 1.55) | 0.114 |  | 0.85 (0.69 to 1.06) | 0.153 |
| Overweight (25.0-29.9) | **1.45 (1.23 to 1.71)** | **<0.001** |  | 1.15 (0.85 to 1.56) | 0.373 |  | **1.81 (1.35 to 2.44)** | **<0.001** |  | **1.48 (1.15 to 1.89)** | **0.002** |
| Obesity (≥30.0) | **2.13 (1.39 to 3.28)** | **<0.001** |  | 1.86 (0.87 to 3.97) ^a^ | 0.111^a^ |  | 2.20 (0.97 to 5.00) ^b^ | 0.061^b^ |  | **2.34 (1.26 to 4.34)** | **0.007** |
| ***Participants without PE*** |  |  |  |  |  |  |  |  |  |  |  |
| Pre-pregnancy BMI group |  | <0.001 |  |  | 0.016 |  |  | <0.001 |  |  | 0.184 |
| Normal weight (18.5-24.9) | ref. | - |  | ref. | - |  | ref. | - |  | ref. | - |
| Underweight (<18.5) | 1.12 (0.99 to 1.26) | 0.075 |  | 1.17 (0.97 to 1.41) | 0.094 |  | 1.20 (0.96 to 1.50) | 0.111 |  | 0.99 (0.80 to 1.22) | 0.907 |
| Overweight (25.0-29.9) | **1.36 (1.17 to 1.58)** | **<0.001** |  | 1.23 (0.95 to 1.57) | 0.112 |  | **1.65 (1.27 to 2.13)** | **<0.001** |  | 1.30 (1.01 to 1.66) | 0.041 |
| Obesity (≥30.0) | **1.72 (1.17 to 2.53)** | **0.006** |  | **2.07 (1.18 to 3.63)** | **0.011** |  | 1.76 (0.87 to 3.58) | 0.119 |  | 1.33 (0.65 to 2.69) ^c^ | 0.436 ^c^ |

Abbreviations: BMI, body mass index; GDM, gestational diabetes mellitus; PE, preeclampsia, OR, odds ratio; CI, confidence interval.

Significant associations and p-values are emphasized in bold font.

ORs were adjusted for maternal age, educational level, and assisted reproduction technology.

^a^ Compared with OR of spontaneous preterm labor including GDM, OR of obesity for spontaneous preterm labor excluding GDM was slightly reduced and the association was not statistically significant.

^b^ Compared with OR of premature rupture of the membranes including GDM, OR of obesity for premature rupture of the membranes excluding GDM was not statistically significant.

^c^ Compared with OR of medically indicated preterm birth including PE, OR of obesity for medically indicated preterm birth excluding PE was significantly reduced and the association was not statistically significant.
