# Supplementary material for: Association of maternal obesity with preterm birth phenotype and mediation effects of gestational diabetes mellitus and preeclampsia: a prospective cohort study
Source: BMC Pregnancy Childbirth. 2022 Jun 1;22:459. doi: 10.1186/s12884-022-04780-2 (PMC9158369; doi:10.1186/s12884-022-04780-2)
Supplement: Supplementary file 2 — Additional file 2: Table S2. The relative risks of preterm birth with or without GDM/PE. [file 12884_2022_4780_MOESM2_ESM.docx]

**Table S2. The relative risks of preterm birth with or without GDM/PE**

|  | GDM | |  | PE | |
| --- | --- | --- | --- | --- | --- |
| BMI | RR (95% CI) ^a^ | P |  | RR (95% CI) ^b^ | P |
| All | 1.43 (1.31 to 1.56) | **<0.001** |  | 7.09 (6.44 to 7.80) | **<0.001** |
| Normal weight (18.5-24.9) | 1.39 (1.25 to 1.54) | **<0.001** |  | 7.39 (6.60 to 8.27) | **<0.001** |
| Underweight (<18.5) | 1.47 (1.13 to 1.92) | **0.004** |  | 6.36 (4.38 to 9.26) | **<0.001** |
| Overweight (25.0-29.9) | 1.30 (1.04 to 1.64) | **0.022** |  | 5.65 (4.47 to 7.15) | **<0.001** |
| Obesity (≥30.0) | 1.10 (0.63 to 1.92) | 0.741 |  | 4.57 (2.68 to 7.79) | **<0.001** |

Abbreviations: BMI, body mass index; GDM, gestational diabetes mellitus; PE, preeclampsia, RR, relative risk;

CI, confidence interval.

Significant p-values are emphasized in bold font.

^a^, the relative risk of preterm birth was calculated by the risk in patients with GDM dividing by the risk

in patients without GDM.

^b^, the relative risk of preterm birth was calculated by the risk in patients with PE dividing by the risk

in patients without PE.
