# Supplementary material for: Association of maternal obesity with preterm birth phenotype and mediation effects of gestational diabetes mellitus and preeclampsia: a prospective cohort study
Source: BMC Pregnancy Childbirth. 2022 Jun 1;22:459. doi: 10.1186/s12884-022-04780-2 (PMC9158369; doi:10.1186/s12884-022-04780-2)
Supplement: Supplementary file 3 — Additional file 3: Table S3. Distribution characteristics of GDM/PE in Different BMI Groups between Preterm Birth. [file 12884_2022_4780_MOESM3_ESM.docx]

**Table S3. Distribution characteristics of GDM/PE in Different BMI Groups between Preterm Birth**

| Parameters | Underweight (<18.5) | Normal weight (18.5-24.9) | Overweight (25.0-29.9) | Obesity (≥30.0) | All | P |
| --- | --- | --- | --- | --- | --- | --- |
| ***Preterm birth cases (n=2768)*** |  |  |  |  |  |  |
| GDM |  |  |  |  |  | **<0.001** |
| No, n (%) | 373 (86.54%) | 1577 (79.13%) | 197 (65.89%) | 27 (60.00%) | 2174 (78.54%) |  |
| Yes, n (%) | 58 (13.46%) | 416 (20.87%) | 102 (34.11%) | 18 (40.00%) | 594 (21.46%) |  |
| PE |  |  |  |  |  | **<0.001** |
| No, n (%) | 413 (95.82%) | 1788 (89.71%) | 246 (82.27%) | 32 (71.11%) | 2479 (89.56%) |  |
| Yes, n (%) | 18 (4.18%) | 205 (10.29%) | 53 (17.73%) | 13 (28.89%) | 289 (10.44%) |  |
|  |  |  |  |  |  |  |

Abbreviations: GDM, gestational diabetes mellitus; PE, preeclampsia.

Significant p-values are emphasized in bold font.
